# The American Journal of Science and the Arts

**Published:** 1851-05

**Authors:** 


### NOTICES OF PAMPHLETS, PERIODICALS, ETC.

ART. IX.—*The American Journal of Science and Arts.* Conducted by Professors B. SILLIMAN, B. SILLIMAN, JR., and JAMES D. DANA, aided in the Departments of Chemistry and Physics by Dr. WOLCOTT GIBBS. New Haven, Conn. March and May numbers.

We have been in the habit of publishing the table of contents of each number of this valuable periodical, but being unable to find room in either our March or April issue for the contents of the March number, we now give the names of the leading papers in the March and May numbers of that able work.

The March number contains papers on the following subjects:

On the Velocity of the Galvanic Current in Telegraph Wires; by B. A. Gould, Jr., in a Report to Professor A. D. Bache, LL. D., Superintendent of the U. S. Coast Survey.

Observations on the Gnathodon beds around the head of Mobile Bay; by Rev. C. S. Hale.

On the Mineral Springs in Canada; by T. S. Hunt.

Whirlwinds produced by the Burning of a Cane-brake; by Alexander Fisher Olmsted, A. M.; with a plate.

Description of a new Graptolite found in the lower Silurian Rocks near the Falls of St. Croix River; by H. A. Prout, M. D.

On the Direction of the Spark from Secondary Currents under the influence of Helices or Magnets; by Professor Chas. G. Page, M. D.

On the Conduction and Distribution of the Galvanic Current in Liquids; by Prof. Chas. G. Page, M. D.

Two Problems by Bäpu Deva Shästri, Native Professor of Mathematics in the Benares College.

On the Vents of Hot Vapor in Tuscany, and their relations to Ancient Lines of Fracture and Eruption; by Sir Roderick Impey Murchison.

On Kirkwood's Law of the Rotation of the Primary Planets; by Prof. Elias Loomis.

On a new Genus of Crustacea in the Collections of the U. S. Exploring Expedition under Capt. C. Wilkes, U. S. N.; by James D. Dana.

Mineralogical Notices.

Notices of Coal in China; by D. J. M'Gowan, M. D.

Abstract of a Meteorological Journal kept at Marietta, Ohio, for the year 1850; by S. P. Hildreth, M. D.

On the different Chemical Conditions of the Water at the Surface of the Ocean and at the Bottom, on Soundings; by Aug. A. Hayes.

On the Limit of Perpetual Snow in the Himalaya; by Lieut. Strachey.

Analyses of the Ashes of certain Commercial Teas; communicated by Prof. E. N. Horsford.

And fifty-three pages of scientific and miscellaneous intelligence, and bibliography.

The May number contains the following papers:

On the Aboriginal Monuments and Relics of New York; by E. G. Squier, A. M.

On the Corrosion of an Alloy, composed of Copper and Silver, in Sea Water; by Aug. A. Hayes.

On the Calculus of Operations; by John Patterson, A. M.

On the Mammoth Cave of Kentucky; by Professor B. Silliman, Jr.

Descriptions of new species of Fishes; by William Prescott, M. D.

Meteorological Observations: Abstract of a Meteorological Journal kept at Beloit College; by S. Pearl Lathrop, M. D.

Miscellaneous Notices; by J. W. Bailey.

On the Chemical Constitution of the Mineral Warwickite; by T. S. Hunt.

On Coral Reefs and Islands; by James D. Dana.

On the Infusoria and other Microscopic Forms in Dust-showers and Blood-rain; by Dr. C. G. Ehrenberg.

Notice of the Geology of the Florida Keys, and of the Southern Coast of Florida; by Prof. M. Tuomey.

On the Law of the Rotation of the Primary Planets; by Daniel Kirkwood.

New Genera of Fossil Corals from the Report by James Hall, on the Palæontology of New York.

Analyses of Pitchstone Porphyry from Isle Royale, and of a Crystal of Phosphate of Lime from Hurdstown, New Jersey; by C. T. Jackson, M. D.

On the Volatility of Phosphoric Acid in Acid Solutions when heated; and on Schmidt's Process for the Determination of Nitrogen; by J. B. Bunce.

On Bromine as a Toxicological Agent; by Henry Wurtz.

On an improved Remontoire Escapement for an Astronomical Clock; by J. Fulton.

And forty pages of scientific and miscellaneous intelligence, and bibliography. B.

